# Superusers’ Engagement in Asthma Online Communities: Asynchronous Web-Based Interview Study

**DOI:** 10.2196/18185

**Published:** 2020-06-23

**Authors:** Anna De Simoni, Anjali T Shah, Olivia Fulton, Jasmine Parkinson, Aziz Sheikh, Pietro Panzarasa, Claudia Pagliari, Neil S Coulson, Chris J Griffiths

**Affiliations:** 1 Asthma UK Centre for Applied Research Institute of Population Health Sciences Queen Mary University of London London United Kingdom; 2 University of Cambridge Cambridge United Kingdom; 3 Asthma UK Centre for Applied Research University of Edinburgh Edinburgh United Kingdom; 4 Asthma UK London United Kingdom; 5 Asthma UK Centre for Applied Research Usher Institute of Population Sciences and Informatics University of Edinburgh Edinburgh United Kingdom; 6 School of Business and Management Queen Mary University of London London United Kingdom; 7 Usher Institute for Population Health Sciences and Informatics University of Edinburgh Edinburgh United Kingdom; 8 School of Medicine University of Nottingham Nottingham United Kingdom

**Keywords:** social networks, eHealth, social media, peer-to-peer support, social support, online health communities, online forums, superusers, leadership, misinformation, asthma, self-management

## Abstract

**Background:**

Superusers, defined as the 1% of users who write a large number of posts, play critical roles in online health communities (OHCs), catalyzing engagement and influencing other users’ self-care. Their unique online behavior is key to sustaining activity in OHCs and making them flourish. Our previous work showed the presence of 20 to 30 superusers active on a weekly basis among 3345 users in the nationwide Asthma UK OHC and that the community would disintegrate if superusers were removed. Recruiting these highly skilled individuals for research purposes can be challenging, and little is known about superusers.

**Objective:**

This study aimed to explore superusers’ motivation to actively engage in OHCs, the difficulties they may face, and their interactions with health care professionals (HCPs).

**Methods:**

An asynchronous web-based structured interview study was conducted. Superusers of the Asthma UK OHC and Facebook groups were recruited through Asthma UK staff to pilot and subsequently complete the questionnaire. Open-ended questions were analyzed using content analysis.

**Results:**

There were 17 superusers recruited for the study (14 patients with asthma and 3 carers); the majority were female (15/17). The age range of participants was 18 to 75 years. They were active in OHCs for 1 to 6 years and spent between 1 and 20 hours per week reading and 1 and 3 hours per week writing posts. Superusers’ participation in OHCs was prompted by curiosity about asthma and its medical treatment and by the availability of spare time when they were off work due to asthma exacerbations or retired. Their engagement increased over time as participants furthered their familiarity with the OHCs and their knowledge of asthma and its self-management. Financial or social recognition of the superuser role was not important; their reward came from helping and interacting with others. According to the replies provided, they showed careful judgment to distinguish what can be dealt with through peer advice and what needs input from HCPs. Difficulties were encountered when dealing with misunderstandings about asthma and its treatment, patients not seeking advice from HCPs when needed, and *miracle cures* or dangerous ideas. Out of 17 participants, only 3 stated that their HCPs were aware of their engagement with OHCs. All superusers thought that HCPs should direct patients to OHCs, provided they are trusted and moderated. In addition, 9 users felt that HCPs themselves should take part in OHCs.

**Conclusions:**

Superusers from a UK-wide online community are highly motivated, altruistic, and mostly female individuals who exhibit judgment about the complexity of coping with asthma and the limits of their advice. Engagement with OHCs satisfies their psychosocial needs. Future research should explore how to address their unmet needs, their interactions with HCPs, and the potential integration of OHCs in traditional healthcare.

## Introduction

### Background

Recent work has suggested that taking part in online communities for people with long-term conditions (LTCs) improves illness self-management [1] and adherence to treatment [2], produces positive health-related outcomes [3-5], facilitates shared decision making with health care professionals (HCPs) [6-8], and may even reduce mortality [9]. There is also evidence that self-management support interventions can reduce health service utilization [10,11]. Participation in online health communities (OHCs) for patients with LTCs can take up part of the health care service demand and indirectly improve access to health care [12]. However, much of this evidence comes from qualitative and observational studies [6,13,14]. Despite a lack of definitive evidence, policymakers are starting to see the potential of OHCs, for example, the Big White Wall, an OHC commissioned by some mental health services in the United Kingdom, Canada, and New Zealand [15]. The Irish health system is piloting closed Facebook groups for smoking cessation [16], whereas the Public Health England Stoptober smoking cessation campaign includes a Facebook group [17] among other initiatives. In the United Kingdom, Facebook is also being piloted by the National Health System Digital (NHS Digital) to improve cancer screening rates, with promising results [18]. This increasing attention to health social media calls for elucidating the mechanisms that make OHC engagement successful in terms of improving self-management [12,19]. Indeed, although some OHCs flourish, many suffer from little or no traffic [20]. The emerging literature investigating mechanisms of effective OHC engagement shows that superusers (ie, users who are in the top 1%-5% in terms of messages posted to the OHC) are key to success. They generate the majority of traffic and create value, so their recruitment and retention are imperative for the long-term success of OHCs [21]. A previous analysis of an online community for people with drinking problems found that common themes for superusers’ engagement included introductions, greetings, general supportive statements, suggested strategies, success stories, and discussion of difficulties [22], showing that superusers reassuringly offer peer support toward behavioral and emotional self-management tasks [23], appropriately leaving to HCPs medical self-management tasks.

To fully understand the unique mechanisms of behavior change through internet-based interventions, collaboration and knowledge transfer between researchers, nonprofit organizations, and private organizations have been recommended [24]. Our network study of peer support in the Asthma UK and British Lung Foundation (BLF) OHCs [25] in collaboration with the platform provider HealthUnlocked has highlighted the key role of superusers. Superusers are distinct from community moderators formally appointed by the platform provider; the number of moderators among the highly active users was negligible [25]. Superusers are a naturally available resource and are responsible for holding successful OHCs together, engage with users with low posting activity, and indirectly contribute to the formation of ties between users. As users become more active within the community, they become more likely to reply to posts than to ask questions. This suggests that superusers gradually become *experts*, providing others with advice and support [2,7,26].

Online superusers could be considered allies of the health care workforce [27,28]. Our work has inspired the development of a new network-based theory of social medical capital, broadly defined in terms of the advantages that any user (patient or caregiver) can gain from participation in OHCs [29].

In this context, strategies to increase superusers’ participation can improve engagement with OHCs [30]. This is attracting growing interest from academics, HCPs, and policymakers. Despite the evidence of superusers’ key role in successful OHCs, little is known about this small but critical population, what motivates them to contribute to the community and stay active over time [22,25], whether they encounter any challenges, how their contribution could be supported in any way, and what would make OHCs safer and more effective. This study set out to understand what motivates superusers to adopt this role and the reciprocal value it offers them. Although the research was pragmatically driven, our interpretation draws on self-determination theory [31], a framework for differentiating intrinsic and extrinsic forms of motivation, to help link the insights to recommendations for health organizations seeking to engage with and utilize the value offered by superusers in their online communities.

### Objectives

Here, we undertook an asynchronous web-based structured interview study of UK superusers previously quantitatively characterized [25], in collaboration with the charity Asthma UK. We additionally aimed to explore superusers’ interfaces with HCPs and their views on HCPs’ potential role in promoting engagement with OHCs and on HCPs’ engagement as OHC participants themselves.

## Methods

### Interview Schedule Development

The interview schedule was developed with questions based on the extant literature, recent work on OHCs [25,32], and informal discussion dated from 2015 to 2018 with 2 superusers, 1 from a stroke OHC previously studied [33] and 1 from Asthma UK OHC.

### Piloting Phase

Piloting was undertaken with 6 superusers recruited through the Asthma UK research operating officer (JP) and OHC moderator. JP emailed the weblink to the study questions and attached to the same email a Microsoft Word document with the interview questions in November 2018. Comments and suggestions were received by JP between November 2018 and February 2019. Superusers’ suggestions improved the clarity of the introductory text and the queries asked. Some questions initially part of the same query were split to make replying easier (ie, questions 3-5 and 7-8), whereas new questions were suggested (ie, questions 10, 13-15, 21, and 24). This process resulted in 10 additional questions. The wording of some questions was also adjusted to make it more neutral to participants.

### Inclusion Criteria

The inclusion criteria were as follows:

- Living with asthma or caring for somebody with asthma
- Having posted to an online asthma community at least one message per week for at least four weeks.

As there is no evidence yet about whether superusers’ posting activity over time is regular or occurring in bursts (eg, when off work due to illness), we opted to be nonspecific about the 4-week period. Therefore, posting activity over any 4 weeks, consecutive or not, at any point in time would qualify participants as superusers.

In this asynchronous web-based structured interview, the definition of superusers is different from the *retrospective* one used in the study by Joglekar et al [25] (ie, top 1% of users characterized by the largest number of posts posted in the community *over the entire observation period of 10 years).* This previous study showed that only about 20 to 30 superusers were active on a weekly basis since 2015. The inclusion criteria for superusers were agreed upon by coauthors and the superusers who took part in the pilot phase. Due to the hypothesized small sample of potential participants available to recruitment [25], no saturation criteria were used to determine the study sample size.

### Participant Recruitment

Of the 17 participants, 16 were recruited by the Asthma UK research operating officer (JP) by email and through an Asthma UK monthly email bulletin to take part anonymously through a SurveyMonkey link [34]. Responses were collected between March and April 2019. A superuser (OF) who is a member of the Asthma UK Centre for Applied Research Patient and Public Involvement (PPI) group [35] was invited and recruited by AD.

### Ethical Approval

The study was approved by the Queen Mary University Research Ethics Committee (ref QMREC2205a). To address the issue of confidentiality around patient information and to avoid this information being known to the research team, superusers were approached only by the Asthma UK staff (JP) and invited to participate. The research team did not have access to personally identifiable information apart from the AUKAR PPI member and coauthor (OF).

### Analysis

We analyzed the text from open questions using inductive content analysis as described by Elo and Kyngas [36]. Two authors (AS and AD) read all responses to familiarize themselves with the data. An initial coding framework with themes and subthemes was developed, which was adjusted as new data were added. This was done for the first 10 individuals and, subsequently, for the additional 7 individuals. Coding was then performed independently by 2 authors (AS and AD) on all data. Coding was discussed until agreement was reached, and the themes were revised as well.

## Results

### Characteristics of the Participants

A total of 17 participants were included in the study (Table 1): 14 were people living with asthma, whereas 3 were mothers of children with asthma. The majority were female (15/17), with an age range of 18 to 75 years (3 out of 17 participants were aged 66-75 years).

Of the 17 users, 10 participated in 2 or more OHCs: 15 out of 17 in Asthma UK HealthUnlocked community and 10 out of 15 in Facebook groups. HealthUnlocked is the platform provider of the Asthma UK online community.

With respect to education, 65% (11/17) had at least an undergraduate degree and 18% (3/17) had a postgraduate degree.

Before taking part in the study, they had been active in OHCs for 1 to 6 years and spent between 1 and 20 hours/week (11 out 17 participants spent ≥2 hour/week) reading and between 1 and 3 hours/week (7 out of 17 participants spent ≥1 hour/week) writing posts.

Self-reported participation increased over time for 14 out of 17 superusers and was linked to wanting to know more about asthma and its treatment in the context of deterioration of asthma or change in medical treatment. Other factors contributing to participation were increased familiarity and interest toward OHC members and improved awareness and knowledge of asthma.

**Table 1:** Participants’ characteristics.

Participant number	Gender	Age range (years)	OHCs^a^ joined	Duration as OHC member (years)	Time spent reading posts^b^ (hours)	Time spent writing posts^b^ (hours)	Number of posts written^b^	Highest level of education
1^c^	Female	36-45	Facebook group (UK Parents of Children with Asthma)	2	5	<1	4-5	Undergraduate degree or similar
2	Female	46-55	Asthma UK	1	1	N/A^d^	0	Postgraduate degree or similar (eg, PhD)
3	Female	66-75	Asthma UK	>1	5	NS^e^	4-10	Undergraduate degree or similar
4	Female	66-75	Asthma UK	2.5	>0.5^f^	NS^e^	Variable^f^	Postgraduate degree or similar (eg, PhD)
5	Female	66-75	British Lung Foundation, Asthma UK	>3	10	2	4	Undergraduate degree or similar
6	Female	46-55	Asthma UK, British Lung Foundation	5	5	<0.5	1	O-levels/ General Certificate of Secondary Education or similar
7	Female	46-55	Asthma UK	5	20	1	5	Prefer not to say
8	Male	46-55	Asthma UK, Facebook	6	1	NS	1	A-level or similar
9	Male	46-55	Asthma UK	0.5	1	0.3	2	Undergraduate degree or similar
10	Female	18-25	Facebook groups, Asthma UK	0.5	1-2	0	0-1	Undergraduate degree or similar
11	Female	46-55	Asthma UK	>3	1-2	1	0-1	A-level or similar
12^c^	Female	36-45	Asthma UK, Facebook group (UK Parents of Children with Asthma)	2.5	2	0.3	4	Undergraduate degree or similar
13	Female	26-35	Asthma UK, HealthUnlocked communities	2.5	6	2-3	15	Postgraduate degree or similar (eg, PhD)
14	N/A	N/A	Facebook group (Bronchiectasis)	<1	0.5	NS	0-5	NS
15	Female	26-35	Asthma UK, Facebook groups	>5	3	1-2	3	Undergraduate degree or similar
16^c^	Female	36-45	Facebook group (UK Parents of Children with Asthma), Asthma UK	2-3	2	0.2	5	Undergraduate degree or similar
17	Female	18-25	Asthma UK, Facebook groups	0.5	3-4	0-1	0-1	A-level or similar

^a^OHCs: online health communities.

^b^Hours of engagement and number of posts refer here to the average week. Some superusers’ engagement may be concentrated over certain time periods.

^c^Superuser is a carer (mother of a child with asthma).

^d^N/A: not applicable.

^e^NS: not stated.

^f^Higher contribution when not well with asthma.

### Themes

Themes and subthemes were generated through content analysis of open-ended questions and are shown in Table 2. Our findings will be articulated into 4 themes:

1. Motivation to engage: Motivation to active participation in OHCs included personal advantage and the desire to help others/being altruistic. Engagement with OHC promoted superusers’ sense of personal control, agency (ie, the actual ability to deal with a task or situation), and self-efficacy (ie, the perceived ability to deal with a task or situation) over their illness, particularly when they adopted the informal role of *wise mentors* wise mentors or supporters to other users. An important reason for people taking part in asthma OHCs was the reward felt by being helpful to other members.
2. Awareness of the limits of peer self-management support: Superusers showed a sense of awareness of what can be dealt with through peer support/advice (ie, behavioral and emotional self-management tasks) and of what instead would require input from HCPs (ie, medical self-management tasks).
3. Challenges: Superusers encounter several challenges, such as dealing with misunderstanding about asthma and treatment and a general sense of responsibility toward other OHC users. Superusers could find themselves under moral pressure to respond to risk-indicating or inappropriate posts and reporting posts to moderators, which could cause at times cognitive stress.
4. Interface with HCPs: Most HCPs were not aware of OHC engagement. Superusers generally felt that HCPs should direct patients to well-trusted and moderated OHCs. Some felt that HCPs themselves should take part in OHCs.

**Table 2:** Themes.

Themes			Subthemes
**Motivation**			
	Seeking information and support	Gaining knowledge about asthma illness and its treatment Validation of own experiences in the context of asthma Feeling less isolated Talking with fellow sufferers Enjoying reading conversations of users one got to know	
	Helping others	Giving advice that could potentially save a life Supporting others with asthma and their carers Making people with asthma and their carers feeling less isolated Show others that living with chronic conditions is not always negative Making sure people with asthma take their disease seriously Disseminating of scientific information	
	Feeling rewarded through helping	Positive feeling when helping others Replacement of role	
	Financial or social recognition not important	Not important: reward is helping others, enjoying interactions Not important: all users shall be equal with equal voice importance Not important: voluntary nature—financial incentives potentially causing misuse Unsure/yes important: recognition of sensible/knowledgeable users	
**Awareness of the limits of peer self-management support**			
	Decisions on posts to reply to	Drawing from personal experience Providing a different point of view	
	Types of support offered	Behavioral and emotional peer self-management support Empowering patients and carers through own experience Signposting to source information and support	
	Medical self-management needs HCPs’^a^ input	Showing appropriate insight of potentially serious medical issues Advising to seek medical help with appropriate urgency	
**Challenges**			
	Top 3 problems and difficulties encountered	Worrying about users struggling with asthma and not seeking medical help as appropriate Misunderstanding, spam, miracle cures, or dangerous ideas Negative tone of some conversations	
	Posts causing superusers’ worries	Religion-based advocacies; derogatory, emotionally challenging posts Offering bad advice or indicating that users have little knowledge about asthma and its gravity	
	Need of additional policies and guidance	Improve awareness of existing policies and guidance for safe engagement with asthma OHCs^b^ Policies and guidance about buying asthma medications on the web Quicker removal of bad posts/advice	
**HCPs and asthma OHCs**			
	HCPs’ awareness of engagement with asthma OHCs	Engagement with asthma OHCs is not discussed during consultations with HCPs Engagement with asthma OHCs is not discouraged by HCPs HCPs’ belief that engagement with asthma OHCs focuses patients on illness and potentially increases their anxiety	
	HCPs’ promotion of engagement with OHCs	HCPs should direct patients with LTCs^c^ to moderated/trusted OHCs Advantage is obtaining behavioral and emotional self-management support that HCPs may not be able to offer Ways of promotion: posters up in the waiting rooms of relevant hospital departments and General Practice (GP) surgeries and face-to-face discussion of OHC information by nurses during asthma clinics	
	Suggestions to reassure HCPs about OHC engagement	Clearer statements about contacting HCPs for medical self-management Improving HCPs’ awareness of benefits of online peer support; robust evaluation of the effects of OHC engagement Readily accessible guidance about keeping safe in social media Clear rules about posting activity; regular, nonintrusive participation of moderators	
	HCPs’ participation in OHCs	Benefits: for the opportunity to get worries and questions addressed, as long as HCPs’ identity is stated Difficulties: potential scrutiny of all posts, limitation of expression of different points of view, lack of sufficient clinical details, and issues with code of conduct of HCP registering bodies	

^a^HCP: health care professional.

^b^OHC: online health community.

^c^LTC: long-term condition.

#### Motivation of Engagement

##### Seeking Information/Support

Motivation to engage with OHCs was linked to personal advantage through gaining knowledge and support for asthma and its treatment:

To learn from others who actually know what it can be like and to learn from their experiences.

N.4

Validation of own experiences in the context of asthma and the feeling of being less isolated were also important factors:

To get validation from others with the same symptoms.

N.17

Having the opportunity to talk with people who live with asthma was considered important:

...many people find comfort and support in such communities that cannot be offered by family members and/or friends that have not experienced the day-to-day living of conditions.

N.14

Reading other users’ conversation was described as a positive experience that increased engagement:

I enjoy the chats with others and reading the dialogue between others, many of whom I’ve got to know.

N.5

##### Helping Others

Altruisms and the benefit of feeling in a position to help others were a significant factor sustaining the motivation to regularly take part in OHCs.

Some even mentioned the potential to save lives:

If my own message or experience could help save a child’s life.

N.1

Most participants talked about the motivation to support others with asthma and their carers to mitigate their sense of social isolation:

…especially when you are supporting parents who are new to dealing with asthma in their child.

N.1

I remember how lost and frustrated and alone I felt. I don’t want other mums who are new to asthma to have those same feelings and struggles.

N.16

...being there to give emotional support when needed.

N.17

Part of the motivation was to show others that living with chronic conditions is not always negative and to offer hope to others:

I wish to show that I can live a normal life with chronic condition.

N.2

Using their knowledge to clear up any confusion about asthma and medications was relevant, as well as making sure people with asthma took their disease seriously and did not rely on social media for queries that needed HCPs’ input:

Trying to make people take their asthma more seriously and not rely on social media for the answers which often don't come and then they end up in hospital.

N.15

Interestingly, a participant mentioned that part of the motivation was to disseminate proper scientific information:

To be helpful and disseminate information, especially scientific information.

N.8

##### Rewards for Online Health Communities Engagement

Participants found helping others a positive experience for themselves. By providing replies to other users’ queries, superusers increasingly acquired confidence and were recognized for their role as community *experts*, which in turn boosted their motivation to further engage in OHCs. For some participants, who were unable to work due to ill health or were retired, taking part in OHCs could work as a replacement of role:

I feel that I can still use the knowledge and skills from my previous work along with my personal experience of asthma.

N.3

I use it far more when I am unwell with asthma.

N.4

##### Financial or Social Recognition Not Important

A question addressed whether the contribution superusers make could be recognized in any way (socially or financially), considering it might help other patients to manage their illness better. Of the 17 participants, 13 replied negatively, 3 were unsure, and only 1 replied positively.

The main reason behind the *no* answer was that reward should come from the awareness of helping others and the fact that social interaction is actually enjoyable.

The motivation to ensure that all users felt equally important to the whole community also played a role:

There is no guarantee that a superuser is any better informed than any other user. Superuser status might make others feel their contributions were not worth making. In my view, the forum should be one where every voice has equal status.

N.9

Other factors against social and financial recognition were the voluntary nature of contributions and the potential misuse of financial incentives:

I do it because I can, if I didn’t want to I wouldn't.

N.16

Some positively saw the social recognition as sensible/knowledgeable users:

Those who are particularly helpful could be recognised with titles, so users know who to contact for issues. Monetary incentives could also be considered, but most people who help online are doing it just to be helpful rather than for any other purposes.

N.10

#### Awareness of the Limits of Peer Self-Management Support

##### Decisions on Posts to Respond to

When asked about what determines their decision to reply to certain posts, participants showed a reassuring awareness of the type of self-management support they were able to offer (ie, emotional and behavioral but not medical tasks):

By drawing in on your own experiences. You soon recognise familiarities with symptoms.

N.1

I choose the ones which I have some relevant knowledge or experience to reply to.

N.3

Some mentioned that they posted replies when they felt they were able to provide a different/unrepresented point of view with respect to the ones already given, which in turn could help others make decisions:

...there may not be another voice in that comment section giving the view I feel, so I may choose to add it.

N.16

Participants’ aim was to empower patients and carers through their own experiences:

I will post a reply from my own experience of asthma gained over 50 years.

N.11

##### Types of Support

The type of support most frequently provided by our respondents was mainly behavioral and emotional. In addition, most participants also mentioned their role in signposting users to source of information and support:

Reassurance, information, sharing my experience of a particular health issue, information about where to go for further advice and information.

N.3

##### Medical Self-Management Needing Health Care Professionals’ Input

Medical self-management was unanimously agreed upon as something that required consultation with HCPs, and all superusers had prior experience of referring other community members to their HCPs:

I never give specific medical advice though.

N.10

Almost every time, the default answer is always to contact your own medical help for guidance.

N.16

#### Challenges

##### Problems and Difficulties

Of the 17 superusers, 9 described problems and difficulties associated with their role in the OHCs (2 were unsure about it, 4 replied no, and 2 did not reply).

The main difficulty described by superusers was the worry they felt regarding other community members who were not successfully managing their asthma and not seeking appropriate medical help:

Members who put their health at risk by not realising how dangerous a situation they are in.

N.17

Other problems described included dealing with misunderstandings, spam, or posts promoting miracle cures or dangerous ideas (eg, buying medicine over the internet). Of the 17 participants, 9 had experience of reporting such posts to moderators:

Spam e mails, folks responding who’ve not understood my posts, prolonged communication.

N.2

People offering “miracle” cures; people not being supportive; going off topic of the original post.

N.14

Some users found it difficult to deal with the negative tone of some conversations, when the underlying aim was to complain:

Some people don't want to take advice and will just complain constantly no matter what you suggest.

N.15

Only 1 user mentioned being trolled once in the past and this being a negative experience. Asthma UK HealthUnlocked community was described as a *good* *forum*.

##### Posts Causing Superusers Worries/Stress

Posts causing superusers worry were about religion-based advocacies and derogatory or emotionally challenging stories. Posts offering bad advice, indicating that users had little knowledge of asthma and its gravity, and revealing a sense of responsibility of superusers to reply to posts and moral pressure toward other OHC users also caused worry and stress.

Moreover, superusers worried about posts from users who were struggling or acutely unwell and subsequently stopped posting or from users who had been chronically struggling with their asthma without seeking professional help:

Those that encourage people with less knowledge to take actions that could put their child in danger/people who are disparaging someone else’s intelligence or understanding of the illness.

N.16

[Posts suggesting that] children can be in respiratory distress.

N.16

##### Suggestions for Policies and Guidance

Of the 17 participants, 8 believed that more policies and guidance should be available for asthma OHCs (2 did not, 4 were unsure, and 3 did not answer the question). In particular, they felt that additional policies and guidance should be introduced on the rules for safe engagement with asthma OHCs and for clarifying when emergency medical advice is needed. Some participants did acknowledge that such policies were already in place, though not all users seemed to be aware of them. A suggestion was made for new users to be encouraged to passively engage and read posts before active engagement:

New members should be encouraged to read without contributing at first. I think all members do this instinctively anyway...joining a social group is rarely instant. Good sites include few risks, made safe by the site rules, moderators and experienced users.

N.5

One participant recommended having policies and guidance about buying medications over the internet.

A number of participants highlighted the importance of quick removal of clearly bad advice so as to develop patient confidence in participation:

Quick removal or “bad” posts (including spam). [Moderators to] Respond to occasional posts (especially if there’s an argument going on in the feed).

N.13

#### Interface With Health Care Professionals

##### Health Care Professionals’ Awareness of Engagement With Asthma Online Health Communities

Most participants’ HCPs (10 out of 17) were not aware of superusers’ engagement with asthma OHCs. Only 3 out 17 participants reported that their OHCs’ involvement was known by their HCPs, whereas 4 out of 17 participants were unsure. Even when the HCPs were aware, this was because superusers mentioned their engagement with OHCs, though they did not discuss it any further:

The only person who knows is my husband.

N.3

Of the 17 participants, 15 stated that they did not believe that HCPs would discourage participation in asthma OHCs:

I think the quality of the BLF and Asthma UK sites is generally accepted. Medics know I’m am open minded and analytical about any information.

N.5

Only 1 participant reported being discouraged by their HCP from engaging with OHCs, and this was linked to concerns about patients becoming focused on illness rather than health and well-being:

They seemed to feel that by engaging with other people in online health forums it focused people on the illness rather than on getting on with life. They seemed to feel that it made people more anxious about their illness rather than provide reassurance, information and support. It seems to me that they were worried that it reinforced an 'illness' mentality. However, my experience is that generally it empowers people to take control of their own lives and make decisions in partnership with their medical carers rather than feel disempowered and uncertain.

N.3

##### Health Care Professionals Promote Engagement With Asthma Online Health Communities

The majority of participants (11 out 17) thought that HCPs should direct patients with LTCs to OHCs, provided they were appropriately moderated and trusted platforms. The remaining 6 out of 17 participants were unsure, though no one felt that HCPs should not promote engagement with OHCs:

Any recommended communities would need to be appropriately vetted/ endorsed by medical professional to ensure their accuracy in terms of medical advice and to keep people safe.

N.10

Respondents offered several specific suggestions about how to promote engagement with OHCs:

There could be posters up in the waiting rooms of relevant hospital departments and GP surgeries. Asthma nurses could inform patients. Ask people how they feel they have benefited from online communities.

N.9

Indeed, a range of advantages arising from the promotion of OHCs by HCPs included obtaining behavioral and emotional self-management support that HCPs may not be able to offer as easily:

For support for people when they get diagnosed, have a really difficult time with their asthma and recovering.

N.11

##### Suggestions to Reassure Health Care Professionals About Online Health Community Engagement

To reassure HCPs about the safety of OHCs, participants felt OHC providers should have clearer statements about contacting HCPs for medical self-management, place more emphasis on the fact that posts from peers come from not medically qualified people, and have a readily accessible guidance about keeping safe in social media. Comments from moderators should be regular and nonintrusive, with strict rules regarding posts:

Healthcare professionals may need to be reassured that any group they signpost is a medically sound one. However, they cannot dictate. It is about mutual respect for the role of the medical professional and the role of an on-line health community.

N.3

Participants felt improving HCPs’ knowledge and awareness of why patients engage with OHCs and the benefits of peer support on LTCs would make them keener to promote OHCs. Evaluation of the impact of engagement in OHCs on patients was also suggested:

[HCPs’ awareness that] online communities are primarily useful for feeling more “normal” with your condition - connecting with others in the same situation.

N.10

##### Health Care Professionals’ Participation in Online Health Communities

When exploring whether HCPs should themselves take part in OHCs, 9 out of 17 participants replied positively, 5 were unsure, 2 were against it, and 1 did not answer the question.

The reasons behind perceiving HCPs’ participation beneficial were the opportunity to get worries and questions addressed. However, as this respondent notes, their participation may be mutually beneficial through learning more about the patient experience of their illness:

Not only could a lot of peoples’ worries and questions be easily answered authoritatively healthcare professionals could gain much knowledge from forums.

N.8

There was a mention of engagement in OHCs as an additional remunerated duty for HCPs:

I think they [HCPs] should be paid to set aside time to monitor forums.

N.8

Most participants felt that HCPs’ participation in OHCs was important as long as their identity was stated:

They should include their medical specialisms in their profiles and understand that there are many viewpoints on some issues.

N.5

Difficulties making participants unsure about HCPs’ participation were potential scrutiny of all posts, limitation of expression from different points of view, and the problem of not knowing the clinical details of users well enough before an appropriate answer could be given:

I feel the community is for those who don't otherwise have a voice and that it would seem too “preachy” to have a medic commenting on every post.

N.9

...it might get difficult (difficult/awkward for medics when dealing with people they know little about).

N.13

Issues with HCPs’ code of conduct and difficulties with HCPs being patients themselves were also expressed:

Difficult. There is a place for it but I think it blurs the lines a little and their code of conduct with their registering body...I think as a healthcare professional who is also a patient they need to be aware of the blurred line between patient and healthcare worker.

N.15

## Discussion

### Principal Findings

This is the first study to provide evidence of superusers’ motivations for engagement in a large nationwide OHC, the challenges they face when interacting with other users, and their interface with HCPs. As the use of social media in health care is increasing, taken together with our previous network study [25], these results provide unprecedented insight on superusers who are key to creating value, driving and sustaining user engagement and contributing to the success of an OHC.

Superusers are both patients with asthma and carers of a wide age range, tend to take part in more than one OHC, and spend considerable time in a role sometimes similar to that of moderators [37]. Reassuringly, they showed awareness of the complexity of coping with asthma and the limits of their advice, provided emotional and behavioral self-management support, and had at times to direct users to HCPs for medical queries. This is an important point as much of the work exploring HCPs’ views of OHCs suggests that they are concerned that inappropriate advice is commonly shared and that community members may not be skilled/reflective enough to realize it.

The superuser role appears to be acquired by users as they deepen their asthma-related knowledge and become accustomed to web-based communication and the dynamics of group-based anonymous interaction [26], turning into *expert patients*, acquiring some of the characteristics of the *second generation of e-patients* [8].

Although the superuser role could be stressful at times, most HCPs were unaware of superusers’ engagement with OHCs and therefore unable to provide support. This is also in contrast with the general agreement among superusers that patient engagement with trusted and thriving OHCs should be promoted within health care. For some, being a superuser could work as a *replacement* of role, as in the case of a retired HCP participant or a participant in working age who is identified as an HCP off work due to asthma.

Superusers who were themselves HCPs raised the issue of the need to develop a code of conduct within their registering bodies to engage with users in OHCs.

It has been suggested that HCPs’ engagement with OHCs could be remunerated as part of HCP duties.

Superusers’ perspectives on what would make OHCs safer and more effective are of interest not only to OHC platform providers but also to policymakers who are increasingly considering leveraging OHCs for health care delivery.

### Strengths and Limitations

There are a number of strengths and limitations to our work, which merit comment. The data we collected from superusers in this study came from an existing and thriving asthma OHC [25,27]. In our previous study, we uncovered the emergence of superusers (or hubs) as the OHC network grew larger. Our findings suggest that users with a disproportionate number of contacts started to emerge only when many users had already joined the network (about 1000). This has important implications for the size of our sample of superusers. Although the absolute number of superusers in this study may appear to be small, it takes a very large-scale network for these superusers to emerge. Thus, our sample size must be gauged jointly with the (large) size of the underlying network to which the superusers belong. Although no saturation criteria were used to determine the study sample size, our qualitative analysis revealed that saturation of emerging themes was reached.

The currently limited literature about superusers in OHCs, the lack of a formal identification of *superuser status* in OHCs, and the a posteriori definition of superusers (ie, superusers as the top 1% active users over a 10-year period [25]) make it difficult to judge the response rate in this study. Our previous work [25] showed the presence of 20 to 30 superusers active on a weekly basis. Although for obvious reasons we could not use the same definition to identify superusers in this study, based on our previous results, the superuser response rate would support the validity of the data presented here.

The study benefited from a superuser piloting phase that face-validated the questions and improved their focus, resulting in additional questions. The study was not designed to test the self-determination theory, which was used as an interpretive lens.

The Asthma UK and Facebook communities are established OHCs (Asthma UK OHC has been operational since 2006) and are moderated and trusted; thus, the results may not extend to other OHCs. Although we cannot confirm superuser sharing of scientific information being always appropriate, in such circumstances, it is likely moderators and other superusers, as seen in this study, would intervene in providing rectification.

Moreover, the self-selective nature of recruitment may have introduced a subjective bias, as less altruistic superusers with different characteristics may not have responded to the invitation.

### Comparison With Existing Literature

Only a handful of studies characterizing superusers in OHCs are present in the literature, and to our knowledge, this is the first direct account of superusers’ motivation to engage in OHCs. Previous work focused on quantifying superusers [21] and their posting behavior [24] using a passive approach. Superusers have been described as mostly female [24], at times, assuming a role similar to moderators [37]. The desire for agency and mastery in asthma patients has been previously described [38]. Engagement with OHCs promoted superusers’ sense of personal control/agency/self-efficacy over their illness, particularly when adopting the informal role of wise mentors or supporters to other users. Interestingly, a recent study indicated that patients gained empowerment through OHCs, which was positively related to patient commitment to the physician and to patient compliance with the proposed treatment [2]. Moreover, there is evidence that users who are high engagers (such as superusers who are themselves patients) exhibit the greatest improvement in patient activation measure (PAM; a measure that captures the extent to which people feel engaged and confident in taking care of their condition) in HealthUnlocked OHCs, even if the average change in PAM across all levels of engagement is not clinically meaningful [39].

### Interpretation of Findings Through the Lens of Self-Determination Theory

**Figure 1:**
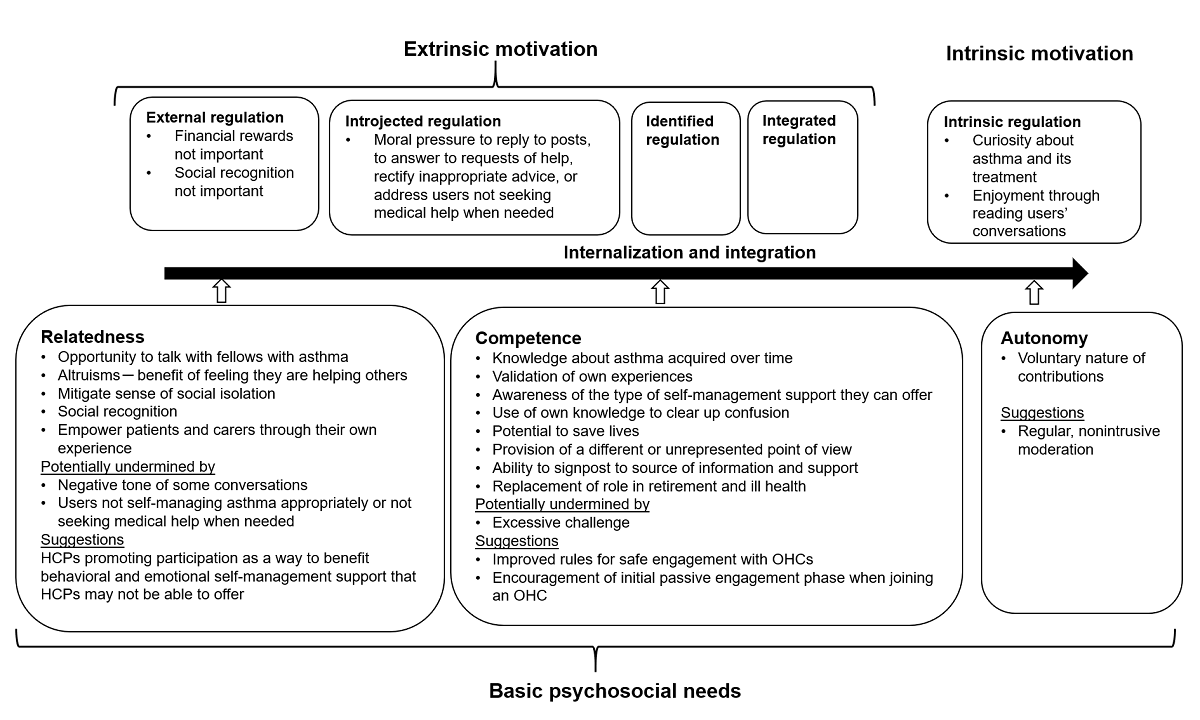
Superusers’ self-determination theory, freely adapted from Ryan and Deci’s theory. Intrinsic motivation constitutes the most autonomous form of motivation and is highly evident in superusers. Such motivation emerges from pure personal interest, curiosity, or enjoyment through engagement with online health communities. The transition from external to intrinsic regulation is promoted by superusers’ fulfillment of the 3 basic psychosocial needs: relatedness, competence, and autonomy. Within the basic psychosocial needs, factors potentially undermining fulfillment and suggestions for improvement are listed. HCP: health care professional; OHC: online health community.

Superusers display high intrinsic motivation to engage with OHCs (Figure 1) [31]. Intrinsically motivated behaviors are carried out for the sake of sheer interest or satisfaction derived from the task. Intrinsic motivation constitutes the most autonomous form of motivation and is highly evident in the participants of this study [31]. Through OHCs’ engagement, they exhibited fulfillment of the 3 basic psychosocial needs: relatedness, competence, and autonomy. With respect to relatedness, superusers described a sense of belonging to the community and a feeling that they mattered to other users. Participants also expressed a sense of mastery (competence), believing in the effectiveness of their ongoing interactions with users within the OHCs. Their behavior is self-endorsed, reflecting autonomy. Superusers are autonomous and wholeheartedly behind their engagement with OHCs. With such strong intrinsic motivation, extrinsic motivation, that is, behaviors that are carried out to obtain outcomes unrelated to the activity itself, such as financial rewards, unsurprisingly, is not particularly relevant. Nevertheless, moral pressure to monitor OHCs, answer to requests of help, rectify any inappropriate advice, or address users not seeking medical help when appropriate were extrinsic motivation factors that at times felt difficult and stressful, needing to be internalized and integrated in their role of superusers.

### Clinical and Research Implications

There is a need to improve clinicians,’ researchers,’ and policymakers’ awareness of superusers. Clinicians could inquire about OHCs’ engagement during consultations with patients with LTCs and offer support to any potential superusers. The first step is establishing a definitive trial to determine whether a primary care intervention specifically aimed at promoting engagement with trusted and thriving OHCs improves the health and well-being of patients with LTCs.

If integration of OHCs proves to be beneficial, given superusers’ potential (ie, 10 superusers can sustain a community of 1000 people) [25,27], campaigns to promote patients with LTCs actively engaging with disease-specific and trusted OHCs may be a way to tackle the demand for behavioral and emotional self-management support in LTCs. Through participation in OHCs, patients who were unable to work due to ill health or retired naturally acquire over time the role of superusers and become a resource to the community, as shown in this study.

Further research should investigate the possible role of HCPs in OHCs based on their monitoring activity and contributions to web-based conversations. Indeed, the Big White Wall [15] and Health Service Executive Facebook [16] are already including HCPs in the delivery of health care.

Further studies of OHC superusers are needed, aimed at addressing their unmet needs and understanding their role as mentors, their learning potential, and how other users within the community learn from them. Using more explicitly self-determination theory approaches can inform the design of new theoretically informed strategies for planning, managing, and sustaining OHCs.

As with the UK NHS face-to-face peer supporters in mental health, the usefulness and development of potential training packages for superusers could be explored.

Superusers expressed the need to improve OHC moderation through quicker removal of harmful posts. This could be achieved by taking advantage of advances in artificial intelligence, which increasingly allow real-time monitoring of OHCs and identifying and *quarantining* posts until review by moderators.

HCPs’ registering bodies may need to develop a code of conduct for HCPs’ participation in OHCs, especially when they take on a superuser role.

These results should be considered in the current increasingly wider uptake of digital skills across populations, with 95% of UK adults being on the internet [40]. In this context, OHCs assume growing potential as vehicles of health and social interventions [41], with the presence of superusers playing a key role in guaranteeing OHC success or failure. The roll out of the NHS app through the National Health Service [42] and initiatives such as the Online Centres Network [43] are working to tackle digital and social exclusion by providing people with the skills and confidence they need to access digital technology. Indeed, 70% of homeless people use social media [44], and the estimated penetration of broadband connection ownership and the tendency to be influenced by web-based content are wider in ethnic minorities [45].

This study offers a novel and fresh perspective on motivation, difficulties, and interaction with HCPs of superusers, a group of patients likely to be key players in the digital health social media landscape.

## Abbreviations

BLF: British Lung Foundation
HCP: health care professional
NHS: National Health System
LTCs: long-term conditions
OHC: online health community
PAM: patient activation measure
PPI: Patient and Public Involvement

## References

[1] Allen C, Vassilev I, Kennedy A, Rogers A (2016). Long-term condition self-management support in online communities: a meta-synthesis of qualitative papers. J Med Internet Res.

[2] Audrain-Pontevia A, Menvielle L, Ertz M (2019). Effects of three antecedents of patient compliance for users of peer-to-peer online health communities: cross-sectional study. J Med Internet Res.

[3] (2015). National Voices.

[4] Mo PK, Coulson NS (2012). Developing a model for online support group use, empowering processes and psychosocial outcomes for individuals living with HIV/AIDS. Psychol Health.

[5] Pendry LF, Salvatore J (2015). Individual and social benefits of online discussion forums. Comput Hum Behav.

[6] Bartlett YK, Coulson NS (2011). An investigation into the empowerment effects of using online support groups and how this affects health professional/patient communication. Patient Educ Couns.

[7] Izuka NJ, Alexander MA, Balasooriya-Smeekens C, Mant J, De Simoni A (2017). How do stroke survivors and their carers use practitioners' advice on secondary prevention medications? Qualitative study of an online forum. Fam Pract.

[8] Duncan TD, Riggare S, Koch S, Sharp L, Hägglund M (2019). From information seekers to innovators: qualitative analysis describing experiences of the second generation of e-patients. J Med Internet Res.

[9] Hobbs WR, Burke M, Christakis NA, Fowler JH (2016). Online social integration is associated with reduced mortality risk. Proc Natl Acad Sci U S A.

[10] Panagioti M, Richardson G, Small N, Murray E, Rogers A, Kennedy A, Newman S, Bower P (2014). Self-management support interventions to reduce health care utilisation without compromising outcomes: a systematic review and meta-analysis. BMC Health Serv Res.

[11] Taylor S, Pinnock H, Epiphanou E, Pearce G, Parke H, Schwappach A (2014). A rapid synthesis of the evidence on interventions supporting self-management for people with long-term conditions: PRISMS - practical systematic review of self-management support for long-term conditions. Health Serv Deliv Res.

[12] De Simoni A, Griffiths CJ, Taylor SJ (2016). Improving access to primary care: can online communities contribute?. Br J Gen Pract.

[13] Ali K, Farrer L, Gulliver A, Griffiths KM (2015). Online peer-to-peer support for young people with mental health problems: a systematic review. JMIR Ment Health.

[14] Taylor J, Pagliari C (2019). The social dynamics of lung cancer talk on Twitter, Facebook and Macmillan.org.uk. NPJ Digit Med.

[15] Harding C, Chung H (2016). Behavioral health support and online peer communities: international experiences. Mhealth.

[16] Facebook.

[17] Facebook.

[18] (2019). NHS Digital.

[19] Craig P, Dieppe P, Macintyre S, Michie S, Nazareth I, Petticrew M, Medical Research Council Guidance (2008). Developing and evaluating complex interventions: the new medical research council guidance. Br Med J.

[20] Bender JL, Jimenez-Marroquin MC, Ferris LE, Katz J, Jadad AR (2013). Online communities for breast cancer survivors: a review and analysis of their characteristics and levels of use. Support Care Cancer.

[21] van Mierlo T (2014). The 1% rule in four digital health social networks: an observational study. J Med Internet Res.

[22] Cunningham JA, van Mierlo T, Fournier R (2008). An online support group for problem drinkers: AlcoholHelpCenter.net. Patient Educ Couns.

[23] Corbin J, Strauss A (1985). Managing chronic illness at home: three lines of work. Qual Sociol.

[24] van Mierlo T, Voci S, Lee S, Fournier R, Selby P (2012). Superusers in social networks for smoking cessation: analysis of demographic characteristics and posting behavior from the Canadian cancer society's smokers' helpline online and StopSmokingCenter.net. J Med Internet Res.

[25] Joglekar S, Sastry N, Coulson NS, Taylor SJ, Patel A, Duschinsky R, Anand A, Evans MJ, Griffiths CJ, Sheikh A, Panzarasa P, De Simoni A (2018). How online communities of people with long-term conditions function and evolve: network analysis of the structure and dynamics of the asthma UK and British lung foundation online communities. J Med Internet Res.

[26] McDonald D, Woodward-Kron R (2016). Member roles and identities in online support groups: perspectives from corpus and systemic functional linguistics. Discourse Commun.

[27] De Simoni A, Taylor S, Griffiths C, Panzarasa P, Sheikh A (2018). NEJM Catalyst.

[28] Selby P, van Mierlo T, Voci SC, Parent D, Cunningham JA (2010). Online social and professional support for smokers trying to quit: an exploration of first time posts from 2562 members. J Med Internet Res.

[29] Panzarasa P, Grffiths CJ, Sastry N, De Simoni A (2020). Social medical capital: how patients and caregivers can benefit from online social interactions. J Med Interent Res.

[30] van Mierlo T, Hyatt D, Ching AT (2015). Mapping power law distributions in digital health social networks: methods, interpretations, and practical implications. J Med Internet Res.

[31] Ryan R, Deci E (2000). Self-determination theory and the facilitation of intrinsic motivation, social development, and well-being. Am Psychol.

[32] De Simoni A, Horne R, Fleming L, Bush A, Griffiths C (2017). What do adolescents with asthma really think about adherence to inhalers? Insights from a qualitative analysis of a UK online forum. BMJ Open.

[33] De Simoni A, Shanks A, Balasooriya-Smeekens C, Mant J (2016). Stroke survivors and their families receive information and support on an individual basis from an online forum: descriptive analysis of a population of 2348 patients and qualitative study of a sample of participants. BMJ Open.

[34] (2018). SurveyMonkey.

[35] Asthma UK Centre for Applied Research.

[36] Elo S, Kyngäs H (2008). The qualitative content analysis process. J Adv Nurs.

[37] Coulson N, Shaw R (2013). Nurturing health-related online support groups: exploring the experiences of patient moderators. Comput Hum Behav.

[38] Pinnock H, Slack R, Pagliari C, Price D, Sheikh A (2007). Understanding the potential role of mobile phone-based monitoring on asthma self-management: qualitative study. Clin Exp Allergy.

[39] Costello RE, Anand A, Jameson Evans M, Dixon WG (2019). Associations between engagement with an online health community and changes in patient activation and health care utilization: longitudinal web-based survey. J Med Internet Res.

[40] Prescott C (2019). Office for National Statistics.

[41] van der Eijk M, Faber MJ, Aarts JW, Kremer JA, Munneke M, Bloem BR (2013). Using online health communities to deliver patient-centered care to people with chronic conditions. J Med Internet Res.

[42] (2020). NHS Digital.

[43] (2020). Online Centres Network.

[44] Guadagno R, Muscanell N, Pollio D (2013). The homeless use Facebook?! Similarities of social network use between college students and homeless young adults. Comput Hum Behav.

[45] (2013). OfCom.

